# Aluminum Adjuvant-Containing Vaccines in the Context of the Hygiene Hypothesis: A Risk Factor for Eosinophilia and Allergy in a Genetically Susceptible Subpopulation?

**DOI:** 10.3390/ijerph15050901

**Published:** 2018-05-03

**Authors:** Todd D. Terhune, Richard C. Deth

**Affiliations:** College of Pharmacy, Department of Pharmaceutical Sciences, Nova Southeastern University, 1382 Terry Bldg, 3200 South University Drive, Fort Lauderdale, FL 33328, USA; rdeth@nova.edu

**Keywords:** allergy, aluminum, asthma, eosinophil, hygiene hypothesis, IgE, vaccination

## Abstract

There are similarities between the immune response following immunization with aluminum adjuvants and the immune response elicited by some helminthic parasites, including stimulation of immunoglobulin E (IgE) and eosinophilia. Immunization with aluminum adjuvants, as with helminth infection, induces a Th2 type cell mediated immune response, including eosinophilia, but does not induce an environment conducive to the induction of regulatory mechanisms. Helminths play a role in what is known as the hygiene hypothesis, which proposes that decreased exposure to microbes during a critical time in early life has resulted in the increased prevalence and morbidity of asthma and atopic disorders over the past few decades, especially in Western countries. In addition, gut and lung microbiome composition and their interaction with the immune system plays an important role in a properly regulated immune system. Disturbances in microbiome composition are a risk factor for asthma and allergies. We propose that immunization with aluminum adjuvants in general is not favorable for induction of regulatory mechanisms and, in the context of the hygiene hypothesis and microbiome theory, can be viewed as an amplifying factor and significant contributing risk factor for allergic diseases, especially in a genetically susceptible subpopulation.

## 1. Introduction

Atopy is a heritable disease associated with a heightened capacity to produce IgE in response to common environmental and food antigens, in which genetic factors confer increased susceptibility to allergic responses, triggered by environmental factors. Vaccination with aluminum-adjuvant-containing vaccines may act as a trigger in such susceptible individuals, and it is recognized that individuals can and do react differently to vaccination [1,2]. Aluminum-containing compounds, primarily aluminum hydroxide (AH), have been widely employed as adjuvants in vaccines to augment the immune response, and the number of recommended aluminum-containing vaccines has greatly increased in the past 25 years. For example, the number of recommended vaccines prior to school entry increased from 10 in 1983 to 32 in 2015, 18 of which contain aluminum adjuvants [3]. There is great interest in the study of vaccines, their components, and the mechanisms by which they interact with and induce immune responses. The new knowledge from these studies can help elucidate the mechanisms by which aluminum-containing-vaccines can act to trigger adverse responses.

Eosinophils are bone marrow-derived granulocytes whose development and differentiation are under the control of several cytokines including interleukin-3 (IL-3), granulocyte-macrophage colony-stimulating factor (GM-CSF), and interleukin-5 (IL-5). Of these cytokines, the biological effects of IL-5 are best characterized and the most potent on eosinophils [4,5]. For example, IL-5 induces terminal maturation of eosinophils, prolongs eosinophil survival, possesses eosinophil chemotactic activity, increases eosinophil adhesion to endothelial cells and enhances eosinophil effector function [6,7]. IL-5 is produced by Th2 cells in response to stimuli such as parasites [8] and viruses [9], or during allergic responses [10]. IL-5 has also been shown to be produced by spleen cells, CD4^+^ T cells, and mast cells in response to aluminum adjuvants [11,12,13,14,15]. In an aluminum hydroxide/ovalbumin-sensitized mouse asthma model, IL-5 deficiency abolished eosinophilia [16]. Therefore, it follows that aluminum adjuvants can induce the production of eosinophils [14,17,18] and eosinophilia [19].

Eosinophilia is defined as a peripheral blood eosinophil count > 500/µL [20] and is associated with conditions such as asthma and atopic diseases, and helminth infections [21]. For instance, atopic asthmatics display a predominance of eosinophils and mast cells [22,23]. The IL-5 gene may play a pivotal role in blood eosinophilia associated with atopic dermatitis (AD) and may contribute to a genetic susceptibility [24]. Recent studies have shown that allergen-challenged atopic asthmatic children exhibit high levels of IL-5 [25,26]. Moreover, according to Toma et al. [27], infants suffering from severe AD show impaired growth, developmental delay, a low serum albumin level, electrolyte disturbances, and have significantly higher number of eosinophils and eosinophilic nuclear lobes, platelets, and total serum IgE level.

The hygiene hypothesis proposes that a reduced exposure to microbes results in immune dysregulation and is associated with the observed increase in allergic diseases [28]. Extending the hygiene hypothesis, the microbiome theory proposes that the gut and lung microbiome composition and interaction with the immune system plays an important role in a properly regulated immune system, and that disturbances in composition are risk factors for asthma and allergies [29]. We propose, in the context of the hygiene hypothesis and microbiome theory, that aluminum adjuvants, especially in a young, genetically susceptible subpopulation, may contribute to an already imbalanced/dysregulated immune system, making them more vulnerable to developing allergic diseases.

This review will examine the contribution of aluminum adjuvants to eosinophilia and allergic diseases, their relationship to helminth infection, the hygiene hypothesis and the emerging microbiome theory, and the adverse outcomes that may arise under those conditions in a genetically susceptible subpopulation of children.

## 2. Aluminum Adjuvants, IL-5 and Eosinophils

BALB/c mice injected with aluminum potassium sulfate (alum) + ovalbumin (OVA) demonstrate an increase in IL-5 production and eosinophils compared to control mice receiving OVA [30]. In another study of alum-injected mice, eosinophils were rapidly recruited to the site of injection, and almost all innate IL-4^+^ cells had staining characteristics of eosinophils [13]. Further, alum-injected mice showed increased levels of IL-5 and the eosinophil chemotactic protein eotaxin, as compared to PBS-injected mice, although eotaxin was shown to be dispensable in eosinophil recruitment following alum immunization, suggesting other pathways may be involved in the eosinophil response to alum [13]. For example, eosinophil recruitment is known to be promoted in part by mast cell-derived IL-5 and histamine [13,31].

Administration of alum increases the number of eosinophils in the peritoneal cavity and in the spleen [13,32]. For example, intraperitoneal (i.p.) injection of female BALB/c and C57B1/6 wild-type (WT) mice with alum induced a population of Gr1^+^ IL-4^+^ cells in the peritoneal cavity and spleen. In the peritoneum, >95% of Gr1^+^ IL-4^+^ cells were eosinophils while in the spleen 50% of the Gr1^+^ IL-4^+^ cells were esosinophils [18]. It was suggested that during responses to alum, Gr1^+^ IL-4^+^ cells play a role in the polyclonal effects on B cells as well as, to some extent, the preclusion of Th1 responses [18].

Another study using BALB/c mice demonstrated long-term activation of bone marrow (BM) eosinophils in BALB/c mice immunized with the alum-precipitated T-cell-dependent antigen phOx coupled to the carrier chicken serum albumin (CSA). These activated BM eosinophils obtained the enhanced ability to support the survival of plasma cells [33]. Alum-only injection produced a rise in the number of eosinophils comparable to that seen with adjuvant plus antigen. However, alum-only administration did not support the long-term survival of eosinophils, as their level fell to almost baseline amounts after 3 weeks [33]. Therefore, whereas injection with alum alone produced temporary induction of eosinophils, alum adjuvant plus antigen induced a lasting activation of eosinophils so that after 60 days these activated esosinophils still produced elevated levels of cytokines [33].

There are several different types of aluminum adjuvants available and used commercially and in research studies. They include Imject^®^Alum, Alhydrogel^®^, and hydrated potassium aluminum sulfate, also known as alum. Traditional alum was originally used in human vaccines. Currently, aluminum hydroxide and aluminum phosphate are the two main types of aluminum adjuvants available and used in human vaccines. Imject^®^Alum is used in experimental settings and is chemically different from both aluminum hydroxide and aluminum phosphate. A comparison study of each type’s ability to induce humoral responses in female C57BL/6 mice injected i.p. found alum was the strongest inducer of antibodies as well as cytokines such as IL-5 and IL-6. Alhydrogel^®^ induced intermediate antibody responses and moderate IL-5 and IL-6 cytokine production, while Imject^®^Alum was the weakest promoter of both antibodies and cytokines [14]. Poor antigen adsorption to Imject^®^Alum was likely the cause of its relatively weak adjuvanticity [14]. With regards to eosinophils, in mice injected with PBS or NP11-CGG alone, B cells and macrophages dominated the lavages and the number of eosinophils, was comparably low [14]. However, the number of eosinophils was significantly increased in all aluminum-immunized mice [14].

Activated eosinophils secrete a broad spectrum of mediators, one of which is the T-cell-activating cytokine IL-4 [6,34]. Enhanced IL-4 production by aluminum-adjuvant-induced eosinophils may induce the production of IL-5 from memory T cells in BM. Increased levels of IL-5 may positively affect the generation of eosinophils and, in addition, it may also prolong their lifetime. It is also important to note that eosinophils modulate peripheral B cell numbers in both mice and humans [35] and can support malignant plasma cell growth [36].

This data from murine studies demonstrates that aluminum adjuvants induce IL-5 and other proinflammatory cytokines, as well as inducing long-surviving eosinophils that contribute to an immunologic response, which in turn may influence other proliferating B cell lineages, potentially promoting non-specific immune responses [35].

## 3. The Immune Response to Aluminum Adjuvants vs. Helminth Infections, and the Hygiene Hypothesis and Emerging Microbiome Theory

### 3.1. Aluminum Adjuvants vs. Helminth Infections

There are similarities between the immune response following immunization with aluminum adjuvants and the immune response elicited by some helminthic parasites, including stimulation of IgE and eosinophilia. Aluminum adjuvants were described by Cooper [17] as good stimulants for Th2 type cell-mediated immune response, especially eosinophils. Like helminth eggs [37], aluminum attracts eosinophils by promoting mast cell-dependent IL-5 production [13]. For example, the Th2-biased immune response to schistosome eggs partly reflects the suppressive effects of egg antigen on dendritic cell (DC) activation and the associated lack of polarizing cytokine IL-12 production by these cells during T cell priming [38]. Similarly, aluminum adjuvants such as aluminum hydroxide and aluminum phosphate inhibit the secretion of IL-12 by DCs [39]. Helminth infection, as with allergy, is typically associated with Th2 polarized immune responses evident by the upregulation of IgE production and eosinophilia in both mice and man, and the expression of inflammation caused by helminth infections can be regulated by the host immune response [40]. Failure of the expression of similar mechanisms among individuals predisposed to allergy may be responsible for the expression of allergic diseases in those individuals [41].

There are clear associations between helminth infection and protection against atopy [42,43]. However, some studies have shown that helminth infections are a risk factor for atopy [42]. It has been proposed that this discrepancy can be explained by the burden and chronicity of the helminth infection; whereas heavy and chronic transmission leads to protection against allergic diseases, helminth infections that happen occasionally and are transient in nature are a risk factor for allergic diseases [28,43,44,45]. For example, in schistosomiasis, chronic exposure to egg antigen occurs in the infected host, and this would be expected to lead to continuous boosting of the Th2 response. As the infection passes into the chronic phase, the magnitude of the immune response to the parasite diminishes [46,47,48]. It has been demonstrated in female CBA/J^k^ mice that the percentage of CD25^+^CD4^+^ cells and the overall levels of Foxp3 mRNA increase significantly during the chronic phase of infection [49], this suggests that an accumulation of regulatory T cells (Treg) cells could account for the modulation of the Th2 response. As reviewed in [50], helminth infections at chronic stages are associated with general immune suppression, and evidence indicates that chronic, but not acute, helminth infection is driving responses that protect against allergic diseases.

Further, a recent study using *H. polygyrus* in six-week-old female BALB/c mice demonstrates that some parasites provide for regulatory mechanisms that alum does not. Infection with *H. polygyrus*, although promoting increased general Th2 inflammation, prevented subsequent allergen-specific eosinophilia and Th2 cytokine production [51]. BALB/c mice were infected with *H. polygyrus* on day 14 then subsequently sensitized by OVA/alum on days 0 and 7 and OVA challenged on days 14 and 16. OVA sensitization and challenge in non-infected mice induced marked airway eosinophilia and high levels of the Th2 cytokines IL-5 and IL-4 [51]. Infection with *H. polygyrus* by itself did not induce airway inflammation, but in infected OVA-sensitized and challenged mice, *H. polygyrus* significantly dampened airway eosinophilia and significantly suppressed IL-5 and IL-4 [51]. In this study it was determined that IL-10 was a requirement for protection against subsequent OVA sensitization with alum, and it was found that the expression of Foxp3 by CD4^+^ cells was increased, as was the percentage of CD4^+^CD25^+^ cells that expressed Foxp3 among cells isolated from the thoracic lymph nodes. This suggests that prior infection with *H. polygyrus* protects against subsequent sensitization via regulatory mechanisms.

In addition, tolerance is driven mainly by the mucosal immune system’s ability to appropriately induce regulatory T cells as opposed to effector T cells [13]. Intraperitoneal injection with alum-bound antigen induced CD4^+^CD25^+^ FoxP3^+^CD45RB^low^ Treg cells, but this route of immunization led to the simultaneous induction of these Treg cells and effector Th2 cells, whereas oral administration of antigen resulted in Treg cell induction only [5]. Importantly, Treg cells induced via the intraperitoneal route were not able to prevent sensitization to the co-administered antigen as opposed to Treg cells induced via the oral route which resulted in tolerance [5].

In summary, it is proposed that aluminum adjuvants, unlike chronic helminth infection, do not support the induction of effective regulatory mechanisms, but are more analogous to acute helminth infection, and in this respect are risk factors for allergic diseases.

### 3.2. The Hygiene Hypothesis and Emerging Microbiome Theory

It is thought that helminths play a role in what is known as the hygiene hypothesis [52]. Diminished exposure to microbes during a critical time in early life, which can regulate the immune system and/or induce Th1 opposing Th2 responses, is the foundation of the hygiene hypothesis [28] and is postulated to have increased the prevalence and morbidity of asthma and atopic disorders over the past few decades, especially in Westernized countries [53]. The hygiene hypothesis has been linked to diminished bacterial and viral infections, leading to a reduction in Th1 responses and, as a result, subsequently weaker Th1 responses and unopposed and stronger Th2 responses [54,55]. However, deviation away from Th1 responses by reduced exposure to microbial products does not sufficiently explain all aspects of the hygiene hypothesis. First, a negative relationship between helminth infections and the prevalence of atopic responses has been noted [56,57,58,59,60], even with the fact that helminth infections strongly induce Th2-type responses, including high levels of IgE and circulating esosinophils and mast cells [44]. Second, Th1-mediated disorders such as inflammatory bowel disease [61], multiple sclerosis [62] and type 1 diabetes [63], have increased together with allergic diseases.

The preceding observations suggest a common underlying mechanism of altered immune responsiveness that may involve Treg cells [56,64,65]. However, other mechanisms have been postulated, such as the blocking hypothesis. The blocking hypothesis states that the high level of total IgE caused by helminth infection is able to block Fcε receptors present on mast cells and thus compete for binding with antigen-specific IgE [42,43]. Further study has shown that the blocking hypothesis cannot be the only mechanism to explain the negative association of helminth infections with allergic disorders [58,66].

More recently, the emerging microbiome theory, which extends the hygiene hypothesis, proposes that the composition of the lung and gut microbiomes influences the risk of asthma and allergies. Importantly, the gut microbiome matures during early life, with its function and composition stabilizing within the first 3 years [67,68,69], helping to maintain a well-regulated immune system. Specific bacterial species may have positive effects on the development of a well-regulated immune system. Certain microbes can control the development of Treg cells via their control over the development of dendritic cells in the gut and/or by directly influencing their development [70,71,72]. Microorganisms of the gut can also contribute to the differentiation of Th1 cells, which in turn can help diminish the Th2 skewing that exists in early life [70].

For example, mice colonized with a mixture of *Clostridium* species demonstrated that early life colonization protected against elevated IgE in adulthood; however, the same bacterial mixture given to adults did not lead to protection [73], affirming the fact that early life is a critical time to establish a properly regulated/balanced immune system. It was also shown the *Clostridium* bacterial mixture induced Treg cell proliferation in the colon and reduced IL-4 in the airways to OVA challenge [73]. Another way microorganisms can have positive immunoregulatory effects, even on remote mucosal sites such as the lung, is through their production of short chain fatty acids. The positive effects of short chain fatty acids are the result of their induction of induced Treg (iTreg) cell populations and their modulation of IL-10 production [74,75,76].

Specific species can also have negative effects. For example, the rate of atopy was increased in neonates who had higher levels of *Clostridium difficile* and a higher *C. difficile* to *Bifidobacteria* ratio in their fecal matter [77], and infants who possessed high levels of fecal *Escherichia coli* developed eczema [78].

Thus, it is likely that an altered microbial burden during childhood results in both the lack of an immune shift away from an early life Th2 dominant immune response and reduced general immune suppression mediated by Treg cells.

**Figure 1:**
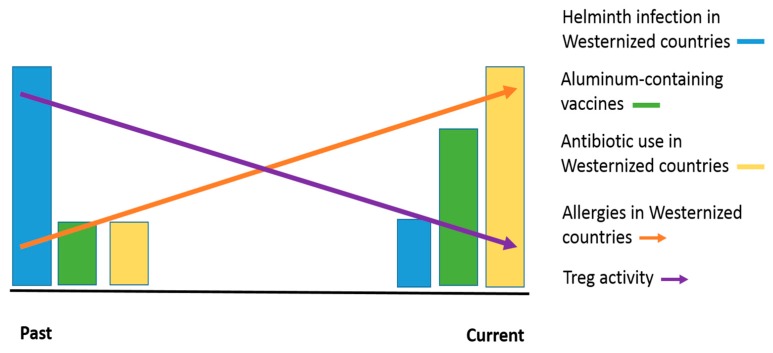
Trends contributing to increased allergic diseases. We propose that increased use of aluminum adjuvants, in light of the hygiene hypothesis and microbiome theory, can promote allergic diseases in a genetically predisposed subpopulation. In the past, helminth infection was more common and was associated with higher Treg activity, while use of aluminum-containing vaccines and antibiotics were relatively low. Over time, helminth infection decreased, while the number of aluminum-containing vaccines and the use of antibiotics increased. These trends could negatively affect Treg activity, as proposed in the hygiene hypothesis and microbiome theory. Considering that aluminum adjuvants may not contribute to an environment conducive to regulatory mechanisms, a subpopulation of young genetically predisposed individuals could be at increased risk for allergic diseases in the current setting.

The above data indicates that immunization with aluminum adjuvants, like helminth infection, induces a Th2 type cell mediated immune response, including eosinophilia, but does not induce an environment conducive to the induction of regulatory mechanisms [51,79]. In the context of the hygiene hypothesis and microbiome theory, aluminum-adjuvant-containing vaccines may be a contributing risk factor for asthma and allergy, especially in a young genetically susceptible subpopulation (Figure 1).

### 3.3. An Alternative View

A recent article suggests, however, that vaccines are unlikely to be causal factors in allergic diseases [80]. This article discusses the hygiene hypothesis and its proposal that the lack of infections in early childhood contributes to a delay in the shift away from early life Th2 dominant immune responses. The article further states that vaccines do not contribute to allergies, in part because vaccines do not prevent the most frequent/commonly acquired childhood infections. It argues that although helminth infections induce strong Th2 immune responses, they are associated with a lower incidence of allergies, and furthermore that conditions that induce strong Th2 responses such as pregnancy are also not associated with higher incidences of allergies. However, this perspective fails to consider the importance of immune regulation in both helminth infection and pregnancy [81]. It also fails to consider aluminum adjuvants specifically and the contributing role they may play. The role for aluminum adjuvants may be more pronounced because of the absence of immune regulation that was historically more prevalent when chronic helminth infection was more common and when microbiomes were better constituted, before changes in the use of antibiotics and changes in diets. The article also argues that diseases associated with Th1 dominate immune responses such as multiple sclerosis occur alongside allergies. Again, the article fails to consider immune regulation, or in the case of multiple sclerosis, the lack of immune regulation [82].

In summary, this perspective fails consider immune regulation in the context of vaccination with aluminum adjuvants in the setting of the hygiene hypothesis and microbiome theory, and further fails to recognize genetic susceptibility that may make some children more vulnerable to allergic diseases in current times.

### 3.4. Aluminum Adjuvants and Immunotherapy

Currently, in the United States, aluminum is not used in immunotherapy, whereas in Europe aluminum is used in immunotherapy as an immune stimulant. As reviewed in [79], aluminum adjuvants in this setting are unlikely to be a determining factor in successful immunotherapy. The induction of CD4^+^ regulatory T cells seems to be associated with suppression after successful immunotherapy [83,84]. Effective Treg responses occur when high allergen doses are given at short time intervals over a long period of time, and these are the determining factors in successful immunotherapy [85].

Treatment with aluminum-containing allergy vaccines contributes to a rise in allergen-specific IgE, at least during the first 6 months [86]; initially, any Tregs induced are unable to regulate IgE production. Aluminum-adjuvant-containing vaccines are typically given over relatively long intervals over a relatively short period of time. Given the differences in protocols and other factors, immunization and immunotherapy that utilize aluminum adjuvants are not comparable.

In summary, it is proposed that successful immunotherapy may be considered to be analogous to chronic helminth infection.

## 4. Susceptibility in a Subpopulation Genetically Predisposed to Allergic Diseases

### 4.1. Features of a Genetically Susceptible Subpopulation

Neonatal mice and humans are thought to have a Th2-biased immune system [87,88]. In human infants, this Th2 skew gradually diminishes during the first 2 years of life in non-allergic individuals [88,89], and this time course correlates well with that of the development of IL-12 production capacity in early life [90]. However, in allergic infants the reverse occurs, with increasing strength of the Th2 response over a similar period [88,89]. In addition, it is well known that Tregs are an important component in the development of normal immune regulation in early life [91].

AD is an inflammatory skin disease that frequently occurs in individuals with a personal or family history of atopic disease. Recent studies show peripheral blood mononuclear cells (PBMCs) from atopic or allergic patients commonly proliferate more extensively and produce more Th2 cytokines in response to allergen stimulation than PBMCs from non-atopic controls [92,93]. Further, when CD25^+^ T cells were depleted from PBMC cultures derived from control subjects these responses were significantly enhanced [92,93]. This indicates that Treg cell- mediated immune suppression occurs in healthy individuals. Further, PBMCs in allergic patients and patients with active hay fever are less sensitive to inhibition by CD4^+^CD25^+^ Treg cells [93,94]. Recent studies suggest that naturally occurring Treg cells and antigen-induced IL-10-producing Treg cells play a role in protecting against human allergic disease [95]. Akdis et al. showed that the number of allergen-specific, IL-10 secreting T cells was significantly increased in non-atopic individuals compared with allergic patients [96]. Other studies similarly demonstrate that IL-10 levels were inversely correlated with the severity of human disease [97,98,99].

Maturing DCs in the presence of IL-10 can produce tolerance-inducing DCs, which can in turn induce IL-10-producing Treg cells (Tr1 cells), playing an important role in the regulation of immune response [100,101]. A recent study analyzed the effect of IL-10-treated bone marrow-derived cells (BMDC) in a Th2 model of allergy using aluminum hydroxide adsorbed OVA for sensitization of BALB/c mice [102]. Treatment of BALB/c mice with OVA-IL-10-DC before and during OVA sensitization did not inhibit OVA-specific cytokine production compared with no transfer of DC [102]. Further, OVA-specific IgE and eosinophil numbers were not inhibited compared to any treatment of BALB/c mice with either OVA-IL-10-DC, IL-10-DC, OVA-DC, or DC alone [102]. These results suggest that Th2 responses induced after sensitization with OVA and aluminum hydroxide are less accessible to inhibition by IL-10 DC.

### 4.2. Aluminum Adjuvants in a Genetically Susceptible Subpopulation

**Figure 2:**
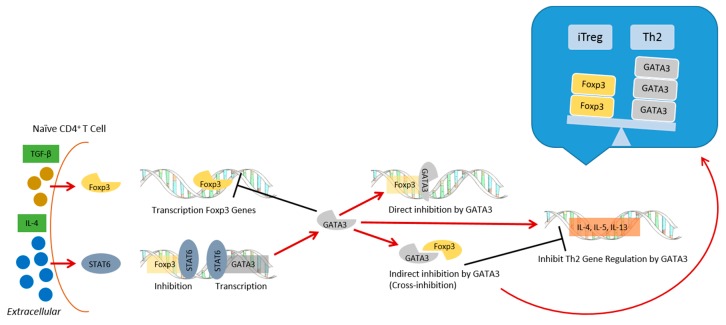
IL-4 competition with TGF-beta. TGF-beta can induce Foxp3, while IL-4 can induce STAT6, which inhibits Foxp3 and promotes GATA3. GATA3 can inhibit Foxp3 directly or indirectly. GATA3 and Foxp3 can bind together and bring about cross-inhibition, thereby creating reciprocal regulation. The relative amounts of GATA3 and Foxp3 could therefore determine the differentiation fate of the cell.

As reviewed in [103], genetic factors in children can have an effect on how they respond to vaccines, including single nucleotide polymorphisms (SNPs) in IL-4 and IL-13 cytokine genes, which are associated with atopy. Aluminum hydroxide, the most commonly used adjuvant in human vaccines, is known to induce Th2 responses in both humans and mice [104]. Human PBMCs treated with aluminum hydroxide produced IL-4 mRNA [105], and it is well known that IL-4 is the most important factor in immunoglobulin class switching to IgE. Further, IL-4 signaling in T cells promotes Th2 differentiation and inhibits Treg development [106]. Signal transducer and activator of transcription 6 (STAT6) is activated by IL-4 in naïve CD4^+^ T cells and increases the expression of the transcription factor GATA-3, which is an important regulator of Th2 cell differentiation [107,108]. Both STAT6 [109] and GATA-3 [110] can bind to the Foxp3 promoter to inhibit Foxp3 gene transcription. This action of IL-4 can contribute to reduced regulation to aluminum adjuvants in a genetically predisposed subpopulation (Figure 2).

Inflammasome activation by aluminum adjuvants has been shown to drive the production of Th2 cytokines such as IL-4, IL-5 and IL-13 [103]. As reviewed in [79], Thymic stromal lymphopoietin (TSLP) has a negative influence on iTreg cell induction. Moreover, high TSLP levels seen in the serum of children with AD compared with normal controls may contribute to susceptibility.

As discussed above, aluminum hydroxide can produce a Th2 response in humans, although it is likely that there is a more mixed Th1/Th2 immune response to aluminum adjuvants in humans [111,112], evidenced by the release of interferon gamma (INF-γ) in response in humans to tetanus toxoid [111]. However, in children who have a family history of atopy, the ability to produce INF-γ appears to be delayed until at least the age of 18 months, and they display a consistently more Th2 skewed DTaP vaccine response [113]. These children are also characterized by Th2 cytokine profiles with high levels of IL-4, IL-5, and IL-13, and may be susceptible to aluminum-containing vaccines that have the potential to induce vigorous Th2 immune responses [103]. Interestingly, a case study describing two children with food allergies showed increased IgE after receiving vaccines, some of which contained aluminum adjuvants. The first child showed an increase in total and in all of her food allergen specific IgE, the second child showed an increase in total and egg-specific IgE [114].

Additionally, allergic reactions to vaccine ingredients such as gelatin have been reported. A 2011 report by the Institute of Medicine, *Adverse Effects of Vaccines: Evidence and Causality*, discusses allergic reactions to gelatin in the MMR vaccine after MMR vaccination. The MMR vaccine does not contain aluminum, but on the current schedule, MMR is given some time after the 3rd dose of diphtheria-tetanus-acellular pertussis (DTaP), which contains both aluminum and gelatin. A study published in 2000 [115] concludes: “We conclude that there was a strong causal relationship between gelatin-containing DTaP vaccination, anti-gelatin IgE production, and risk of anaphylaxis following subsequent immunization with live viral vaccines which contain a larger amount of gelatin.” Although this does not prove that the aluminum in DTaP is directly responsible, this points to the possibility of a dysregulated Th2 immune response after DTaP in some individuals.

**Figure 3:**
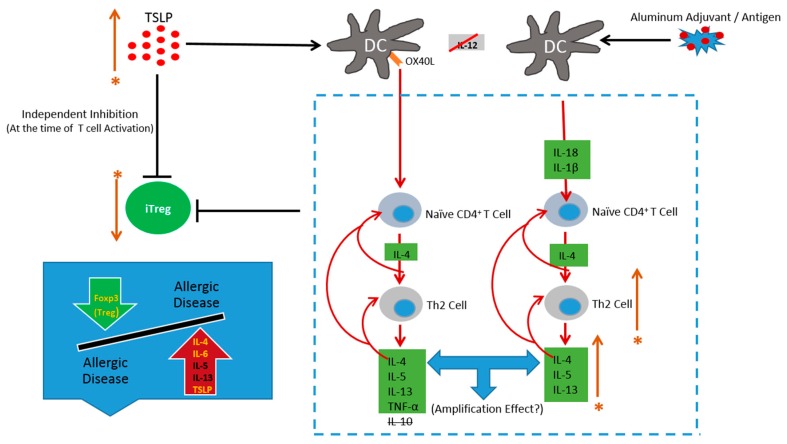
Genetic predisposition. High levels of thymic stromal lymphopoietin (TSLP) and Th2 cytokines such as IL-4, IL-5 and IL-13 have been observed in subjects with allergic diseases, associated with impaired Treg function. Further, an increased Th2/Treg ratio has been reported in patients with moderate to severe asthma. TSLP can drive induction of inflammatory Th2 cells that release IL-4, IL-5, IL-13, and TNF-alpha, but not the regulatory cytokine IL-10. Thus it is important to consider that immunization with aluminum adjuvants may have a stronger than optimal amplification effect, with a further increase in the Th2/Treg cell ratio, resulting in long-term immune dysfunction. * Indicates genetic predisposition.

Aluminum adjuvant-containing vaccines by their nature do not adequately activate Treg cells, leaving open the possibility of strong Th2-biased immune responses, to which the above described genetically susceptible population may be more prone [79] (Figure 3). Thus, the most commonly used adjuvant in vaccines for humans, aluminum hydroxide, may be an important contributing factor to the rise in the occurrence of allergies in Western societies [116]. Moreover, a subpopulation may be more vulnerable to aluminum adjuvants due to hereditary factors, causing an imbalance between Treg and Th2 cells [79].

In summary, it can be proposed that aluminum-containing vaccination in a genetically predisposed subpopulation, in the context of the hygiene hypothesis and microbiome theory, may significantly increase the risk of allergic disease.

## 5. Differences between Mice and Humans

Although the structure of the immune system of mice and humans is quite similar, there are differences in both innate and adaptive responses [117]. The examples below are not an exhaustive list of differences, but act as examples of differences that need to be considered when using data from mouse studies and applying them to humans.

During studies in mice, aluminum adjuvants are typically given via i.p. injections which allow for easy recovery of cells and measurement of cytokines, but the normal route of vaccination in humans is i.m. [111]. When BALB/c mice were injected i.m. with 50 μl of a mixture containing 1.7 mg/ml aluminum hydroxide adjuvant and 200 μg/ml ovalbumin, neutrophils accumulated early followed by macrophages and then eosinophils and MCHII^+^ cells [118]. As mentioned above, mice injected i.p. with different types of aluminum adjuvants responded with IL-5, IL-6 and eosinophils. Mice respond to proteins with alum by producing Th2 isotypes. This indicates aluminum induces a Th2 response in mice, however in humans, responses to aluminum can be mixed [111,112].

In mice, IL-4 induces IgG1 and IgE, in humans IL-4 induces IgG4 and IgE. Also, mouse B cells do not respond to IL-13, whereas in humans IL-13 induces B cells to class switch to IgE [119]. Further, in mice, IL-10 is considered a Th2 cytokine, though in humans Th1 and Th2 cells can produce IL-10 [120].

In summary, the differences noted above require caution when interpreting data in mice studies and applying the data to humans.

## 6. Conclusions

The hygiene hypothesis states that a reduction of a Th1 stimulus and general immune suppression due to the reduced burden of microbes in Westernized societies results in stronger, unopposed Th2 responses and reduced Treg cell immune suppression. Further, the microbiome theory extends the hygiene hypothesis by proposing that the microorganisms of the gut and lung interact with, and help to maintain, a well-regulated immune system, and disruptions in the composition of the microbiome are risk factors for asthma and allergies.

Early life, when the immune system is skewed towards a Th2 response, is a critical time to start establishing a properly balanced immune system. This is also the time when young children start to receive aluminum-adjuvanted vaccines. Mice models of allergy use aluminum as adjuvants to sensitize the animal to an antigen, this indicates an impaired regulatory response in those models. Further, case and other studies have shown an increase in total and food allergen-specific, and vaccine antigen IgE in children after receiving aluminum-adjuvanted vaccines. This indicates that aluminum-adjuvanted vaccines can have an effect on non-target antigens. In present times, when immune regulatory factors are changing, as indicated by the hygiene and microbiome hypotheses, young immune systems, especially those in a genetically predisposed subpopulation, may be more vulnerable to the risk of immune dysfunction from environmental factors such as aluminum-adjuvanted vaccination.

The reviewed data on aluminum adjuvants supports the possibility that aluminum-containing vaccination may be an amplifier of the hygiene hypothesis and microbiome theory, and thus a contributing factor to the increase in allergic disease, especially in a genetically predisposed young subpopulation.

However, further study is needed to determine if indeed a genetic predisposition to atopy makes a subpopulation more vulnerable to aluminum-adjuvanted vaccines. Therefore, any changes from the recommended vaccine schedule to withhold or postpone vaccination needs to be made with respect to the currently known risks and benefits.
